# Pediatric Arachnoid Cysts: A Comprehensive Systematic Review of Clinical Features and Therapeutic Approaches

**DOI:** 10.3390/jcm14196866

**Published:** 2025-09-28

**Authors:** Paula Espinosa Villagomez, Mario S. Hinojosa-Figueroa, Jose E. Leon-Rojas, Gustavo Ignacio Rivas-Martínez, Alcy R. Torres

**Affiliations:** 1 NeurALL Research Group, Quito 170157, Ecuador; paulapalisespinosa@hotmail.com (P.E.V.); mariohinojosaf.0711@gmail.com (M.S.H.-F.); 2 Escuela de Medicina, Universidad Internacional del Ecuador, Quito 170411, Ecuador; 3 Cerebro, Emoción, Conducta (CEC) Research Group, Escuela de Medicina, Universidad de las Américas (UDLA), Quito 170124, Ecuador; 4 Facultad de Ciencias Exactas y Naturales, Universidad Nacional de Asunción, Lorenzo 111421, Paraguay; grivas@facenuna.edu.py; 5 Department of Pediatrics, Division of Pediatric Neurology, Boston Medical Center, Boston University School of Medicine, Boston, MA 02118, USA; artorres@bu.edu

**Keywords:** arachnoid cyst, subarachnoid bleeding, pediatric population, trauma, sports

## Abstract

**Background/Objectives:** Subarachnoid cysts in children, while often linked to trauma, can also rupture spontaneously without any apparent injury. Their etiology remains complex, involving congenital, traumatic, and infectious factors. This article explores the risks, pathophysiology, and management strategies related to spontaneous rupture in pediatric cases reported in the literature through the means of a systematic review. **Materials and Methods:** A systematic review of Web of Science, Scopus, PubMed and the Virtual Health Library (BVS, for its acronym in Spanish) was conducted; the online software Ryyan was used to manage the references and conduct the filtering process. The National Heart, Lung, and Blood Institute (NHLBI) quality assessment tool was used to assess bias for each type of study. **Results:** We analyzed the data of 101 articles; in total we found that 331 pediatric patients with arachnoid cyst were diagnosed with intracranial hemorrhage and 1030 patients had an unruptured arachnoid cyst. The most common cyst diameter was between 5–7 cm in the bleeding group vs. 3–4.5 cm in the non-bleeding group. A head trauma trigger was identified in 36.25% of cases of bleeding and 10.6% were sports related. Most of the hemorrhages were subdural, followed by a mixed pattern between subdural and intracystic. In both groups the arachnoid cyst was mostly located in the middle cranial fossa in the left side. The bleeding arachnoid cysts were mostly treated with surgery, but conservative treatment was also effective; the outcome was good in the majority of cases. **Conclusions**: Further research is required to elucidate the pathophysiological mechanisms underlying hemorrhage associated with arachnoid cysts in the pediatric population. Nevertheless, upon identification of an arachnoid cyst, neurosurgical follow-up is warranted. Bleeding tends to occur only in the presence of high-risk features and can be precipitated by traumatic events.

## 1. Introduction

Arachnoid cysts (ACs) are cerebrospinal fluid (CSF)-filled, intra-arachnoidal lesions that constitute approximately 1% of all intracranial mass lesions; they are typically classified as either primary (congenital) or secondary (acquired) in origin and are most frequently discovered incidentally through neuroimaging [1]. Primary ACs are believed to result from developmental anomalies during the embryological formation of the arachnoid membrane between the 5th and 13th gestational weeks, possibly due to the failure of fusion or duplication of the leptomeninges [1]. Secondary ACs may arise following trauma, infection, hemorrhage, or neurosurgical intervention, resulting in arachnoid adhesions and loculated CSF spaces [1].

**Figure 1:**
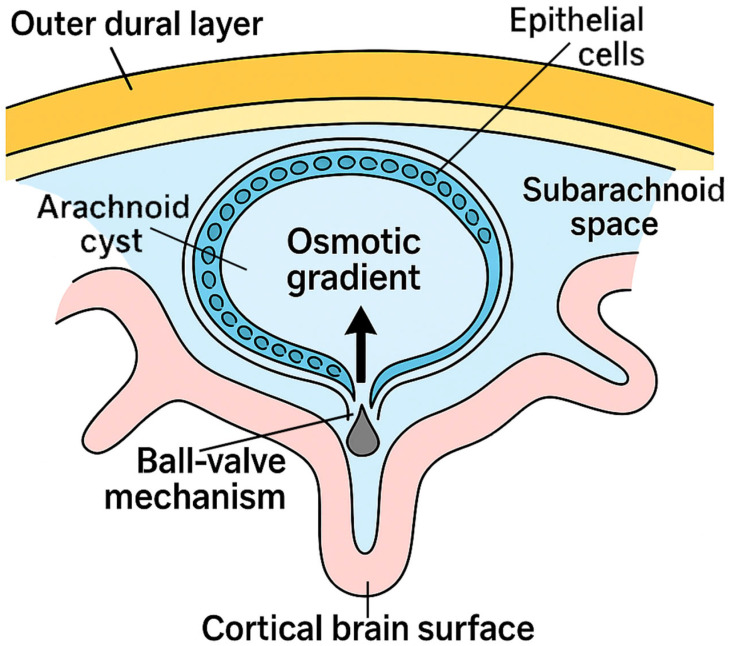
Arachnoid Cyst Pathogenesis showcasing accepted mechanism such as osmotic gradients, ball-valve mechanisms and epithelial-like cells secretion.

The pathogenesis of ACs involves several key mechanisms including active fluid secretion by epithelial-like cells, establishment of osmotic gradients between the cyst and surrounding CSF, and ball-valve mechanisms whereby CSF enters the cyst through one-way communication and becomes trapped, leading to gradual expansion (Figure 1) [2]. While most ACs remain asymptomatic and stable over time, larger or strategically located cysts can exert mass effects, compress adjacent neural structures, or obstruct CSF circulation, thereby producing clinical symptoms such as headache, seizures, or hydrocephalus [2].

A critical yet rare complication of arachnoid cysts is intracystic or subdural hemorrhage, particularly in cysts located in the middle cranial fossa and in young male patients [3]. The predominant theory involves the stretching and rupture of bridging veins traversing over the cyst dome, particularly in the context of minor head trauma [3]. Additionally, the fragility of microvasculature within or adjacent to the cyst wall, compounded by fluctuations in intracystic pressure or trauma-related hyperemia, may contribute to spontaneous or traumatic hemorrhage [3]. The resulting hemorrhages can manifest as subdural hematomas, intracystic hemorrhages, or, less frequently, subarachnoid hemorrhage, potentially leading to acute neurological deterioration [3].

The pediatric population requires a specialized focus in arachnoid cyst research and management given their particularities and differences from adult populations. Children have distinct anatomical features including thinner skulls, larger head-to-body ratios, and ongoing brain development that may influence both cyst formation and rupture risk [3]. The developing brain’s increased plasticity may allow for better adaptation to slowly growing cysts, but rapid changes from hemorrhage can be particularly devastating in this population [2,3]. Furthermore, pediatric patients have different injury mechanisms, with falls and sports-related activities being more common than the motor vehicle accidents typically seen in adults. The long-term implications of arachnoid cysts in children are also more significant given their extended life expectancy and potential for decades of neurological impact [2,3].

Understanding the structural and hemodynamic vulnerabilities of arachnoid cysts is essential for clinicians, particularly in pediatric cases presenting with neurological symptoms or in trauma-prone populations. The phenomenon of spontaneous rupture in the absence of apparent head injury raises critical questions regarding the pathophysiology of arachnoid cysts in pediatric patients and the potential implications for diagnosis, treatment, and long-term outcomes. This article explores the current understanding of arachnoid cysts in children, examining both traumatic and non-traumatic etiological factors, and discusses the clinical risks and management strategies associated with hemorrhagic complications.

## 2. Materials and Methods

Our review follows the recommendations of the Preferred Reporting Items for Systematic reviews and Meta-Analyses (PRISMA) 2020 guidelines and its protocol has been registered in PROSPERO (CRD420251077473).

### 2.1. Eligibility Criteria

Our inclusion criteria encompassed articles reporting on arachnoid or leptomeningeal cysts, whether ruptured or unruptured, and those with or without associated hemorrhage in pediatric populations under 18 years of age. Studies published in English or Spanish, regardless of the year of publication, were considered, including those that reported management strategies or did not specify treatment approaches. Exclusion criteria comprised studies focusing on spinal cysts, adult populations (18 years or older), publications in languages other than English or Spanish, and secondary research such as systematic reviews, meta-analyses, letters to the editor, opinion pieces, or any non-primary research articles.

Spanish and English were chosen as selection criteria given that, first, our research team possessed native or near-native proficiency in both languages ensuring an accurate interpretation of nuanced clinical details; and second, resource constraints prevented the hiring of professional scientific translators for additional languages.

### 2.2. Information Sources and Search Strategy

We queried the most relevant biomedical databases for our study focus, including Web of Science, Scopus, PubMed, and the Virtual Health Library (BVS, for its acronym in Spanish), up to 4 October 2024. Searches were supplemented by manual screening of reference lists from included articles to identify any additional studies that met the eligibility criteria. Studies were included if they involved pediatric patients with arachnoid cysts, had no secondary comorbid conditions, and documented a history of cyst rupture. The screening process was carried out using the Rayyan platform, applying two consecutive filtering phases to determine the final set of eligible studies. Risk of bias for each included article was assessed using the National Heart, Lung, and Blood Institute (NHLBI) Study Quality Assessment Tools according to the corresponding study design. A detailed summary of the search strategy is provided in the Supplementary Material.

### 2.3. Selection Process

Two blinded authors independently reviewed the titles and abstracts of all the articles, after deduplication, against the aforementioned eligibility criteria; if any discrepancies were identified, a third author weighed in until mutual consensus was achieved. Afterwards, in a similar fashion, the remaining articles were further assessed by reading the full-text. Articles that successfully passed the process were then scrutinized for relevant information that was recollected into an Excel spreadsheet.

### 2.4. Data Collection Process and Data Items

Data extraction was performed independently by the reviewers, with any discrepancies resolved through discussion and consensus. Information was collected and organized in a structured Excel spreadsheet that included the following variables: article identifying information (DOI, authors, year of publication), participant demographics (age, sex), cyst characteristics (location, side, and diameter), and type of associated hemorrhage (subdural hematoma, intracystic hemorrhage, or both). The nature of the hemorrhage was further classified as acute, subacute, or chronic. Additional data items included history of trauma or sports-related injury, type of study (case report, case series, cohort, or case–control), imaging modalities used (CT, MRI, or other), and the surgical intervention performed (craniotomy, shunt placement, burr hole, fenestration, or unspecified). Clinical management strategies (conservative or surgical) and patient outcomes were also recorded.

### 2.5. Risk of Bias Assessment

We assessed risk of bias in the included studies using the National Heart, Lung, and Blood Institute (NHLBI) quality assessment tool—namely the Quality Assessment of Controlled Intervention Studies Tool, the Quality Assessment Tool for Observational Cohort and Cross-Sectional Studies, the Quality Assessment of Case–Control Studies tool, and the Quality Assessment Tool for Case Series Studies—for evaluation of experimental, cohort, cross-sectional, case–control, and case series studies, respectively.

The risk of bias in the studies identified as case reports or case series was systematically assessed using the tool developed by Murad et al. (2017), titled “Methodological Quality and Synthesis of Case Series and Case Reports” [4]. This instrument provides a structured framework to evaluate key methodological domains, including selection, ascertainment, causality, and reporting, thereby ensuring a consistent and rigorous appraisal of the internal validity and applicability of such descriptive studies.

Two authors independently applied the respective tool to each included study and recorded the answers for every question. There were three possible answers: yes, no, or other (cannot determine or not applicable). Discrepancies in bias assessment were resolved through a systematic approach: first, both reviewers discussed their scoring rationale for each discrepant item; second, they re-examined the original study together to clarify ambiguous methodological details; and third, when consensus remained elusive, the senior author provided a final determination based on established quality assessment principles. We then calculated a percentage for every study based on the number of yes out of the total number of questions. We classified every study into three categories: minimally low risk if the percentage of “yes” was 80% or higher, moderately low risk if the percentage was between 50 and 79%, and high risk if the percentage was less than 50%.

## 3. Results

**Figure 2:**
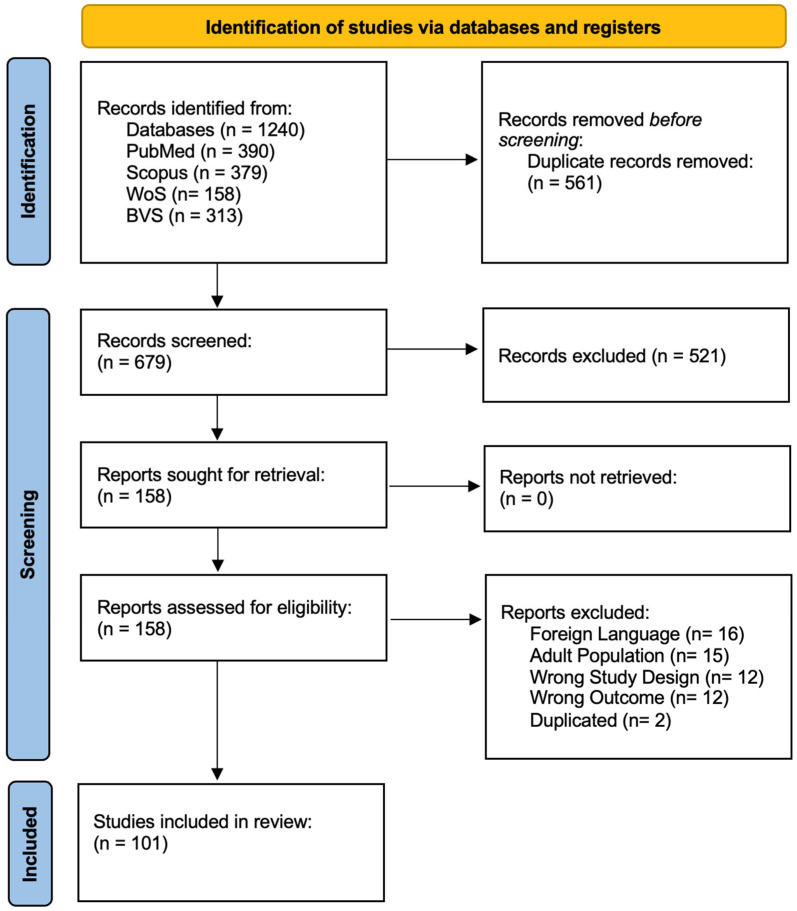
PRISMA flowchart showcasing the filtering process.

Our search yielded a total of 1240 papers. After eliminating duplicates, we were left with 679 unique articles; after the filtering process we selected a total of 101 papers that met all the inclusion criteria, encompassing various study designs, with a particular emphasis on case report studies, representing 83.2% (84 articles), there were also 13 cohort papers (12.9%), 3 case series and 1 case–control study; the full filtering process is presented in Figure 2. Most of the studies had a moderately low risk of bias (Table 1).

### 3.1. Bleeding or Ruptured Arachnoid Cysts

We included a total of 1361 pediatric patients, of which 331 were diagnosed with arachnoid cysts complicated by intracranial hemorrhage [3,5,6,7,8,10,11,12,13,14,15,16,17,18,19,20,21,22,23,24,25,26,27,28,29,30,31,32,33,34,35,36,37,38,39,40,41,42,43,44,45,46,47,48,49,50,51,52,53,54,55,56,57,58,59,60,61,62,63,64,65,66,67,68,69,70,71,72,73,74,75,76,77,79,80,82,83,84,86,87,88,89,90,91,92,93,94,95,96,97,98,99,100,101,102,103,104]. The mean age at diagnosis for these types of complicated cysts was 8.73 years, indicating a predominance of cases in the pediatric and preadolescent population. There was a marked male predominance, with 79.15% of the cases occurring in males, compared to 20.85% in females. Regarding the cyst diameter, reported in 14 studies with bleeding cysts (n = 110), the diameter varied between 5 and 7 cm in 94.55%; it was greater than 7 cm in 0.9% and smaller than 5 cm in 4.55%, suggesting a tendency toward larger cysts in cases that presented with hemorrhagic complications [10,11,12,20,35,38,55,56,91,92,93,97,99,104]. Table 2 presents a summary of the characteristics reported in the literature comparing bleeding/ruptured vs. non-bleeding pediatric arachnoid cysts.

**Figure 3:**
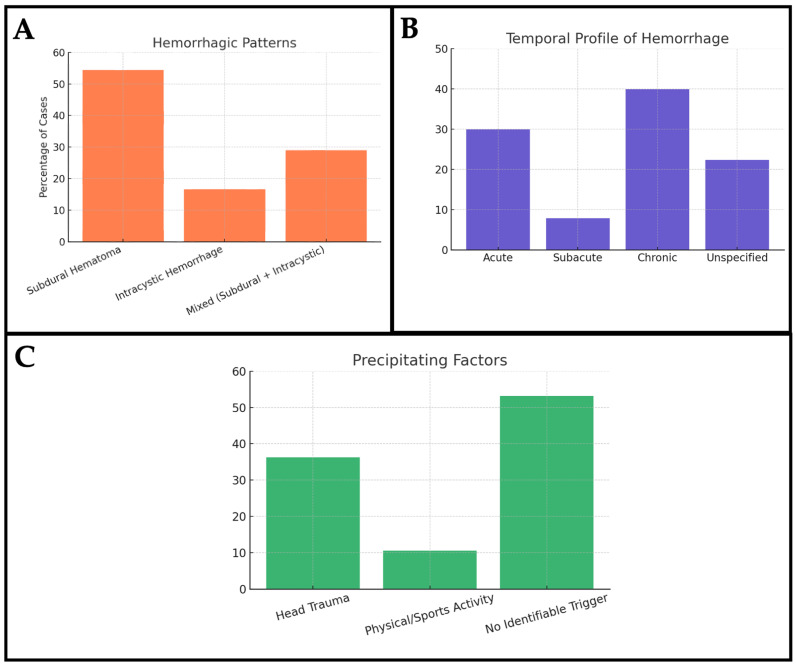
Bar charts summarizing hemorrhagic characteristics in patients with arachnoid cysts. (**A**) shows the distribution of hemorrhagic patterns; (**B**) shows the temporal profile of hemorrhage; and (**C**) shows the frequency of the reported precipitating factors.

In terms of hemorrhagic patterns (Figure 3A), subdural hematoma was the most frequently reported, accounting for 54.4% of the cases [5,8,10,12,14,16,17,18,19,21,22,24,25,27,28,30,31,32,34,35,37,38,41,42,44,45,46,47,48,49,51,53,54,55,57,60,62,63,64,68,69,70,72,73,74,75,76,80,82,83,84,86,87,88,89,91,92,93,94,96,97,98,100,102,104]. Intracystic hemorrhage was observed in 16.6% of patients [20,23,51,56,64,65,69,72,77,92], and a mixed pattern, involving both subdural and intracystic bleeding, was reported in 29% of the cohort [3,6,7,11,13,15,22,26,29,32,33,34,36,39,40,43,49,50,51,52,55,59,61,67,69,79,84,88,90,93,94,95,96,99,101,102,103,104]. With regard to the temporal profile of the bleeding, acute hemorrhage was identified as the most prevalent, in 29.9% of cases [5,12,25,28,31,33,34,37,41,45,51,53,55,63,66,67,69,75,88,93,98,99,100,101,103,104], followed by subacute in 7.85% [5,6,7,8,17,22,25,27,46,52,59,74,80,89,90], and chronic bleeding in 39.88% [3,5,7,8,10,14,15,19,21,24,26,29,32,34,35,36,39,42,43,44,47,49,50,51,53,55,60,61,62,64,68,69,70,72,76,79,80,82,83,84,86,87,88,89,91,92,94,96,97,102]. In the remaining cases, the temporal pattern of the hemorrhage was either unspecified or not reported (Figure 3B). Finally, a precipitating factor was documented in a significant proportion of cases. A history of head trauma was present in 36.25% of patients [3,8,10,12,13,14,16,17,18,20,22,23,24,25,26,27,29,30,31,32,33,34,35,36,43,44,45,46,47,48,49,50,55,62,63,64,65,66,67,68,70,71,72,73,76,77,79,80,84,88,89,91,93,94,95,96,98,99,101,102], and physical or sports-related activity was identified as a contributing factor in 10.6% of cases [3,10,18,20,21,24,25,26,29,32,34,35,38,43,44,47,50,51,61,71,76,80,87,89,91,93,96,98,101,102]. Notably, more than half of the patients (approximately 53%) had no identifiable external trigger, supporting the possibility of spontaneous hemorrhage associated with arachnoid cysts, even in the absence of mechanical stress (Figure 3C).

Neuroimaging played a central role in the diagnosis of these cases. Out of a total of 331 patients, 274 (82.77%) underwent imaging studies, while 57 (17.22%) did not report a specific type of imaging used. Among those with imaging, only 3 patients (0.9%) had an angiography [17,27]. The remaining 271 patients (81.9%) received either computed tomography (CT) or magnetic resonance imaging (MRI), with 240 (72.5%) undergoing CT [3,5,6,7,8,9,10,12,13,14,15,16,17,18,19,20,21,22,23,24,27,28,29,30,31,32,33,35,36,37,39,41,42,43,44,45,46,47,48,50,53,56,58,59,60,61,62,63,64,65,66,67,68,69,70,71,73,74,75,80,82,83,84,87,88,89,91,92,93,94,95,97,98,99,100,101,102,103,104] and 162 (48.9%) undergoing MRI [3,8,10,12,13,16,20,22,25,26,28,29,31,32,33,35,37,40,44,45,49,52,53,54,56,57,58,59,64,65,66,67,68,69,70,71,72,76,79,82,83,84,86,88,89,90,91,94,102,103,104].

**Figure 4:**
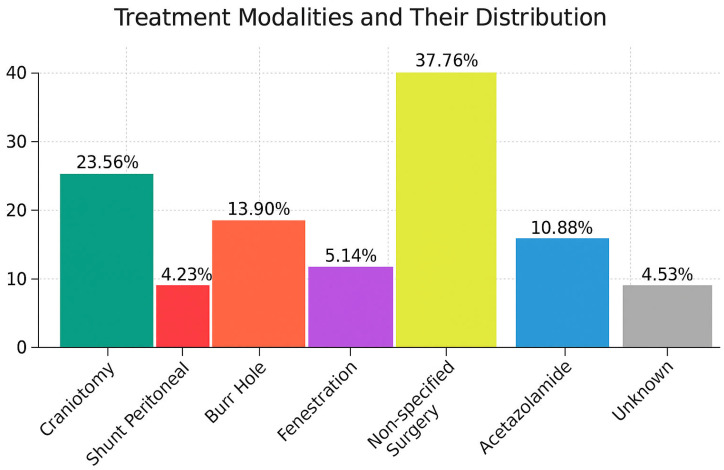
Distribution of treatment modalities among pediatric patients with hemorrhagic arachnoid cysts.

Surgical intervention was the predominant therapeutic approach, performed in 84.59% of cases. Craniotomy was the most frequently performed treatment modality (23.56%) [5,13,14,15,17,18,19,20,21,22,23,25,29,30,31,32,34,36,40,47,49,55,59,61,62,63,67,68,73,74,76,80,82,83,84,85,86,88,91,100,101,102,103], followed by burr hole surgery [5,6,7,8,9,10,20,24,27,32,34,42,43,44,46,54,58,60,64,71,72,73,87,93,95,98,99], fenestration [3,7,38,39], and ventriculoperitoneal shunt placement [7,34,75,89]. In 37.76% of cases, the surgical procedure was not clearly specified, or the treatment modality was not even mentioned (4.53%). A non-surgical (conservative) management strategy was employed in 10.88% of patients, primarily involving the use of acetazolamide, a carbonic anhydrase inhibitor commonly used to reduce cerebrospinal fluid production [16,22,33,34,35,37,41,45,48,51,52,53,56,57,65,66,77,104]. The frequencies of the treatment modalities are shown in Figure 4.

**Figure 5:**
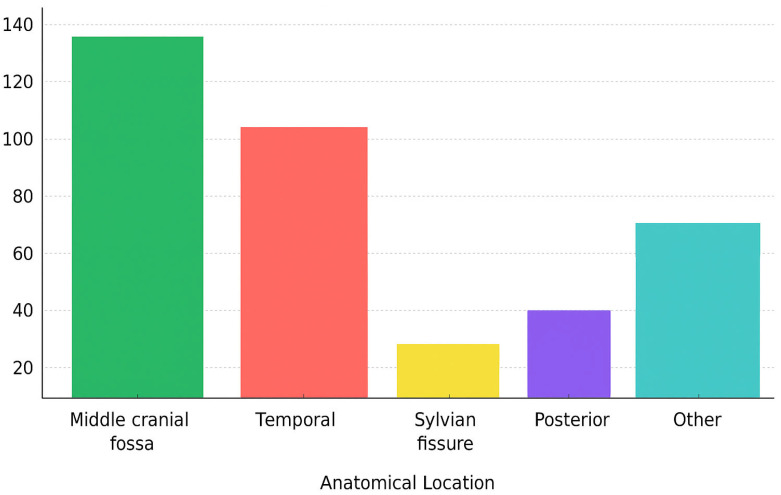
Absolute frequencies of anatomical locations of bleeding arachnoid cysts.

Anatomical analysis of cyst localization revealed that the middle cranial fossa was the most frequently involved site, reported in 73.12% of cases [8,12,14,20,22,25,26,30,31,32,33,35,39,40,41,42,43,51,52,53,54,59,60,62,64,66,69,70,71,72,73,74,76,96,104]; of these, 29.31% were located specifically in the temporal lobe [3,6,7,10,15,16,21,23,27,29,34,36,37,38,43,44,46,47,49,55,58,63,68,73,80,84,89,92,93,94,95,97]. Additional locations included the Sylvian fissure 4.83%, the posterior fossa 1.20%, and other miscellaneous intracranial sites 20.85% [5,11,13,17,18,19,20,24,26,28,30,32,45,49,50,51,55,56,57,61,65,67,69,70,77,79,82,83,86,87,88,90,91,97,98,99,100,101,102,103]. Most lesions were located on the left side (55.59%) [6,7,8,12,15,16,17,18,20,21,23,24,25,26,27,28,29,30,31,32,33,34,35,37,40,43,44,48,49,50,52,54,55,56,58,59,60,61,64,66,68,69,70,71,72,73,76,80,82,83,84,88,89,91,92,93,94,95,96,97,100,102,103,104], followed by the right side (28.70%) [3,7,10,13,14,19,20,22,25,26,32,34,35,36,38,39,41,42,46,47,48,49,53,55,57,62,63,64,65,70,72,73,74,79,80,83,86,87,89,90,93,94,97,98,99,101,104]; a smaller proportion of cases involved unknown laterality 13.60% [5,49,51,69,77,89], while middle and bilateral presentations were rare, accounting for only 1.20% and 0.91% of cases, respectively [7,11,45,67,70,74]; Figure 5 showcases the absolute frequency of bleeding cysts by anatomic location.

Taken together, these findings underscore the clinical significance of arachnoid cysts located in the middle cranial fossa, particularly in male pediatric patients, given their association with hemorrhagic events. Subdural hematoma emerged as the predominant type of bleeding, with a chronic course being the most observed temporal pattern. While trauma and physical activity were relevant triggers in a subset of patients, a large proportion of cases occurred without an identifiable external cause, raising the possibility of intrinsic cyst vulnerability. These observations further support the importance of early diagnosis and proactive clinical monitoring, especially in cases involving large cysts in high-risk anatomical locations.

### 3.2. Non-Bleeding Arachnoid Cyst

Out of the 15 studies included, only 4 specifically reported on patients with arachnoid cysts who had no history of hemorrhage or rupture. These 15 studies accounted for a total of 1030 patients diagnosed incidentally, without bleeding events [3,9,20,26,48,49,51,62,78,80,81,85,92,97,104]. The mean age of this subgroup was 6.98 years, with a predominance of males (76.12%) compared to females (23.88%). The cyst diameter was only reported in 471 individuals, of which a diameter between 3 and 4.5 cm was reported in 98.9% and a diameter greater than 4.5 cm was reported in 1.1% [3,20,51,97].

Computed tomography (CT) was performed in 689 patients [3,20,48,62,78,80,81,85,92,97,104], while magnetic resonance imaging (MRI) was utilized in 479 cases [3,9,20,26,49,85,104]. Notably, 434 patients underwent both CT and MRI, highlighting the complementary use of these modalities in clinical evaluation. In 296 cases, the specific imaging modality was not reported. Regarding potential symptom-triggering factors, head trauma was identified in 7.57% (78 cases) [20,48,81,85,92], and sports-related activities were noted in 19.03% (196 cases) [9,51,81]. However, most cases 73.4% (756 patients) did not specify any identifiable trigger for symptom onset, suggesting that many diagnoses may have been incidental or due to nonspecific clinical presentations.

Among the 1030 cases, cysts were most frequently located on the left side 39.22% [3,20,26,48,62,92,97,104] and right side 20.00% [20,26,48,62,92,97,104], while a significant proportion lacked specification 38.74% [9,26,49,51,78,80,81,85]. Only a minority were reported in the midline 2.04% [26,97], and no bilateral cysts were identified. Regarding anatomical distribution, the temporal region was the most affected site in 51.26% [3,26,49,62,97,104], followed by other less specific locations in 30.87% [9,20,26,51,78,80,81,85,97] and the middle cranial fossa in 16.31% [20,26,51,80]. Rare locations included the Sylvian fissure 1.07% [48] and posterior fossa 0.49% [26].

## 4. Discussion

Currently, there are no standardized protocols or evidence-based guidelines for the clinical management of arachnoid cysts in routine practice. The aim of this systematic review is to enhance the understanding of the clinical presentation and natural history of arachnoid cysts in the pediatric population, thereby facilitating more informed clinical decision-making. To date, this is the most comprehensive systematic review focused specifically on pediatric arachnoid cysts, synthesizing data from 101 studies encompassing a total of 1361 patients.

### 4.1. Clinical Features

The prevalence of arachnoid cysts in the pediatric population is around 2.6%, with greater than 90% being asymptomatic [105]. They are most commonly diagnosed in this population due to increased neuroimaging in early life. While most cysts remain asymptomatic and stable, their potential for expansion, rupture, or bleeding has been documented in the case reports and series compiled in this review [3,5,6,7,8,10,11,12,13,14,15,16,17,18,19,20,21,22,23,24,25,26,27,28,29,30,31,32,33,34,35,36,37,38,39,40,41,42,43,44,45,46,47,48,49,50,51,52,53,54,55,56,57,58,59,60,61,62,63,64,65,66,67,68,69,70,71,72,73,74,75,76,77,79,80,82,83,84,86,87,88,89,90,91,92,93,94,95,96,97,98,99,100,101,102,103,104].

Our findings demonstrate that hemorrhagic arachnoid cysts predominantly affect males (79.15% vs. 20.85% females), with a mean age of 8.73 years at diagnosis [3,5,6,7,8,10,11,12,13,14,15,16,17,18,19,20,21,22,23,24,25,26,27,28,29,30,31,32,33,34,35,36,37,38,39,40,41,42,43,44,45,46,47,48,49,50,51,52,53,54,55,56,57,58,59,60,61,62,63,64,65,66,67,68,69,70,71,72,73,74,75,76,77,79,80,82,83,84,86,87,88,89,90,91,92,93,94,95,96,97,98,99,100,101,102,103,104]. The predominant hemorrhagic pattern was subdural hematoma (54.4%), which aligns with the proposed mechanism of bridging-vein rupture over the cyst dome [106]. Notably, chronic hemorrhages comprised 39.9% of cases, suggesting that many hemorrhagic events may progress insidiously with subtle clinical presentations such as headache or behavioral changes [82,88].

Differential diagnoses should be carefully considered, particularly in atypical locations or presentations. For example, intradiploic pseudomeningocele, an abnormal CSF collection within the diploic space, often post-surgical, can mimic arachnoid cysts on imaging. A recent case report underscores this possibility from pediatric posterior cranial fossa surgery [107]. It is vital to assess surgical history and bone architecture and use advanced imaging to distinguish these entities early in the diagnostic process.

### 4.2. Risk Factors

Several key risk factors emerged from our analysis, though causal relationships must be interpreted cautiously given the predominance of case reports and observational studies. Cyst size represents a significant risk factor: bleeding cysts most often measured between 5–7 cm, while non-bleeding cysts most commonly measured 3–4.5 cm. This supports a previously reported ~5 cm threshold for increased hemorrhagic risk [20]. In comparison, non-bleeding cysts also reported sports and head trauma as potential triggers to symptomatology in 19.03% and 7.57% of cases, respectively. These findings raise an important distinction, as head trauma appears to be the factor most closely associated with AC bleeding and not necessarily sports participation itself. This is further supported by the aforementioned case–control study that found a significant association between head trauma within the prior 30 days and AC bleeding, with an OR of 25.1 (95% CI, 4.0–∞) [20].

Concerning the location, the majority of non-bleeding cysts were located in the middle cranial fossa, representing 67.57% of individuals, specifically in the temporal region in 51.26%. Similarly, bleeding cysts were located in the middle cranial fossa in 73.12% of cases and specifically in the temporal area in 29.31%. These coincide with previous values reported in literature updates [3].

Anatomical location also appears to influence bleeding risk, with middle cranial fossa cysts accounting for 73.12% of hemorrhagic cases. The predilection for left-sided involvement (55.59% vs. 28.70% right side) in bleeding cysts may reflect asymmetric brain development patterns or differential vulnerability of bridging veins, though this observation requires further investigation. Trauma history was documented in 36.25% of hemorrhagic cases and sports-related activity in 10.6% of cases, but importantly more than half of the hemorrhagic cases had no identifiable external trigger, supporting the possibility of spontaneous rupture related to intrinsic cyst features rather than external trauma [108].

### 4.3. Pathophysiological Mechanisms

The pathophysiology of arachnoid cyst hemorrhage involves complex interactions between anatomical vulnerability and hemodynamic factors. The predominant mechanism involves stretching and eventual rupture of bridging veins that traverse the cyst dome, particularly in middle cranial fossa locations where venous anatomy creates greater susceptibility [3]. The cyst wall itself may harbor fragile microvasculature that becomes increasingly vulnerable with cyst expansion. Intracystic pressure fluctuations (e.g., CSF pulsatility or positional changes) may contribute to vessel wall stress and eventual rupture.

The ball-valve mechanism that drives cyst expansion may also create intermittent pressure spikes that cause hemorrhage predispositions. Additionally, the developing brain’s rapid growth during childhood may place additional stress on already stretched bridging veins, explaining the pediatric predilection for hemorrhagic complications [82,88].

### 4.4. Management Approaches

Surgical intervention was the predominant therapeutic approach in hemorrhagic cases (84.59%), with craniotomy being the most frequently employed technique (23.56%) [3,5,6,7,8,10,11,12,13,14,15,16,17,18,19,20,21,22,23,24,25,26,27,28,29,30,31,32,33,34,35,36,37,38,39,40,41,42,43,44,45,46,47,48,49,50,51,52,53,54,55,56,57,58,59,60,61,62,63,64,65,66,67,68,69,70,71,72,73,74,75,76,77,79,80,82,83,84,86,87,88,89,90,91,92,93,94,95,96,97,98,99,100,101,102,103,104]. However, conservative management proved effective in about 10.88% of patients, often with acetazolamide to reduce CSF production and intracystic pressure [3,5,6,7,8,10,11,12,13,14,15,16,17,18,19,20,21,22,23,24,25,26,27,28,29,30,31,32,33,34,35,36,37,38,39,40,41,42,43,44,45,46,47,48,49,50,51,52,53,54,55,56,57,58,59,60,61,62,63,64,65,66,67,68,69,70,71,72,73,74,75,76,77,79,80,82,83,84,86,87,88,89,90,91,92,93,94,95,96,97,98,99,100,101,102,103,104].

The diversity of surgical techniques employed (craniotomy, burr-hole drainage, fenestration, shunt placement) reflects the lack of standardized treatment protocols and the need for procedure selection based on specific clinical circumstances. Future research should focus on developing evidence-based algorithms for treatment selection to optimize outcomes while minimizing intervention-related risks [3,5,6,7,8,10,11,12,13,14,15,16,17,18,19,20,21,22,23,24,25,26,27,28,29,30,31,32,33,34,35,36,37,38,39,40,41,42,43,44,45,46,47,48,49,50,51,52,53,54,55,56,57,58,59,60,61,62,63,64,65,66,67,68,69,70,71,72,73,74,75,76,77,79,80,82,83,84,86,87,88,89,90,91,92,93,94,95,96,97,98,99,100,101,102,103,104].

### 4.5. Clinical Outcomes

Outcomes in hemorrhagic arachnoid cysts were predominantly favorable, with only one death reported among 331 hemorrhagic cases [3,5,6,7,8,10,11,12,13,14,15,16,17,18,19,20,21,22,23,24,25,26,27,28,29,30,31,32,33,34,35,36,37,38,39,40,41,42,43,44,45,46,47,48,49,50,51,52,53,54,55,56,57,58,59,60,61,62,63,64,65,66,67,68,69,70,71,72,73,74,75,76,77,79,80,82,83,84,86,87,88,89,90,91,92,93,94,95,96,97,98,99,100,101,102,103,104]. The effectiveness of both surgical and conservative approaches indicates that treatment decisions can be individualized without compromising patient safety. Long-term neurological outcomes appear favorable in most cases, though systematic follow-up data were limited in the predominantly retrospective studies included in our review. The development of prospective registries with standardized outcome measures would significantly enhance understanding of long-term prognosis and optimal management strategies [3,5,6,7,8,10,11,12,13,14,15,16,17,18,19,20,21,22,23,24,25,26,27,28,29,30,31,32,33,34,35,36,37,38,39,40,41,42,43,44,45,46,47,48,49,50,51,52,53,54,55,56,57,58,59,60,61,62,63,64,65,66,67,68,69,70,71,72,73,74,75,76,77,79,80,82,83,84,86,87,88,89,90,91,92,93,94,95,96,97,98,99,100,101,102,103,104].

Regarding sports participation, our findings do not support blanket restrictions for children with incidentally discovered arachnoid cysts. While trauma represents a documented risk factor, the majority of hemorrhagic events occurred spontaneously, and the overall risk remains low. Sports participation should not be universally restricted without individualized risk assessment considering cyst size, location, and specific activity-related trauma risk. The well-established benefits of physical activity in childhood development must also be considered [109,110].

### 4.6. Limitations 

Our systematic review has several important limitations that should be acknowledged when interpreting the findings. First, the majority of included studies were case reports and small case series, which are inherently prone to selection, reporting, and publication biases. These study designs often lack systematic follow-up, control groups, and standardized outcome measures, limiting the generalizability of findings and precluding robust statistical analysis. Second, there was substantial heterogeneity in clinical, radiological, and surgical data reporting across studies, preventing formal meta-analysis or development of stratified risk models. Key variables such as cyst size, imaging protocols, duration of follow-up, and patient outcomes were inconsistently described, reducing the precision of our synthesis. Third, geographic and publication bias likely influenced our results, as case reports of complicated or unusual presentations are more likely to be published than routine cases. Our language restriction to English and Spanish publications may have excluded relevant studies from other regions, potentially limiting the global applicability of our findings. Fourth, the lack of prospective data significantly limits our ability to establish causal relationships, particularly regarding the role of trauma or physical activity in cyst rupture. The predominantly retrospective nature of included studies introduces recall bias and prevents systematic evaluation of risk factors over time. Fifth, the heterogeneity of reporting standards across studies and time periods prevented formal meta-analysis and limited our ability to generate pooled estimates of important clinical parameters. Standardized reporting guidelines for arachnoid cyst research would significantly enhance future systematic reviews and meta-analyses.

Finally, hemorrhagic or complicated cysts were likely overrepresented in our sample due to publication bias favoring clinically significant cases, potentially leading to overestimation of complication rates and limiting insight into the natural history of uncomplicated lesions. This limitation underscores the need for population-based studies and prospective registries to better characterize the true clinical spectrum of pediatric arachnoid cysts.

## 5. Conclusions

This systematic review provides the most comprehensive synthesis to date of pediatric arachnoid cysts with and without hemorrhagic complications. Our findings highlight that while these cysts are often asymptomatic, a significant subset, particularly those located in the middle cranial fossa and of larger size, may present with hemorrhagic events, most commonly chronic subdural hematomas. Male sex, larger cyst diameter, and trauma history were recurrent features in hemorrhagic cases, although more than half lacked an identifiable trigger, suggesting a possible role for intrinsic cyst vulnerability. Neuroimaging, particularly MRI and CT, plays a central role in diagnosis, and surgical management remains the predominant treatment approach for complicated cysts. However, a select proportion of cases may be safely managed conservatively, underscoring the need for individualized clinical decisions.

Importantly, while trauma is a recognized risk factor, sports participation should not be universally restricted without individualized risk assessment. The overall rarity of hemorrhagic complications must be balanced against the well-established physical and psychosocial benefits of exercise in childhood. Future prospective studies are needed to better characterize the natural history of these lesions, identify reliable predictors of hemorrhagic risk, and develop evidence-based guidelines for monitoring and management. A multicenter registry incorporating standardized data on imaging, treatment, and outcomes could be instrumental in advancing our understanding of these lesions.

## Figures and Tables

**Table 1:** Risk of bias.

Author (Year)	Study Type	Level of Bias
Aydln, S. (2022) [5]	Cohort	Moderately low
Aydogmus, E. (2019) [6]	Case report	Moderately low
Balestrino, A. (2020) [7]	Case series	Moderately low
Bilginer, B. (2009) [8]	Case report	Moderately low
Bonow, R. (2017) [9]	Cohort	Moderately low
Borni, M. (2023) [10]	Case report	Moderately low
Bose, T. (2011) [11]	Case report	Moderately low
Canty, K. (2021) [12]	Case report	Moderately low
Cao, H. (2024) [13]	Case report	Moderately low
Cappelen, J.(1986) [14]	Case report	Moderately low
Chandra, V. (2015) [15]	Case report	Moderately low
Choong, C. (1998) [16]	Case report	Moderately low
Chung, M. (2020) [17]	Case report	Moderately low
Clavel, E. (1983) [18]	Case report	Moderately low
Cole, W. (1996) [19]	Case report	Moderately low
Cress, M. (2013) [20]	Case–control	Moderately low
Cullis, P. (1983) [21]	Case report	Moderately low
Datta, D. (2024) [22]	Case report	Moderately low
De, K. (2002) [23]	Case report	Moderately low
Domenicucci, M. (2009) [24]	Case series	Moderately low
Donaldson, J. (2000) [25]	Case–control	Moderately low
Eidlitz, T. (2014) [26]	Cohort	Moderately low
Ergüngor, M. (2006) [27]	Case report	Moderately low
Eustace, S. (1992) [28]	Case report	Moderately low
Furtado F. (2019) [29]	Case report	Moderately low
Galassi, E. (1986) [30]	Case report	Moderately low
Gelabert-González, M. (2002) [31]	Case report	Moderately low
Gregori, F. (2020) [32]	Case report	Moderately low
Hagan, M.(2021) [33]	Case report	Moderately low
Hall, F. (2021) [34]	Case series	Moderately low
Hamidi, M. (2021) [35]	Case report	Moderately low
Hara, H. (1984) [36]	Case report	Moderately low
Pinto, V. (2021) [37]	Case report	Moderately low
Hopkin, J. (2006) [38]	Case report	Moderately low
Iaconetta, G. (2006) [39]	Case report	Moderately low
Iglesias, A. (2006) [40]	Case report	Moderately low
Inoue, T.(1987) [41]	Case report	Moderately low
Jafrani, R. (2019) [3]	Case report	Moderately low
Kang, J. (2015) [42]	Case report	Moderately low
Kawanishi, A. (1999) [43]	Case report	Moderately low
Kertmen, H. (2012) [44]	Case report	Moderately low
Kizilkiliç, O. (2003) [45]	Case report	Moderately low
Krishnan, P. (2013) [46]	Case report	Moderately low
Kulali, A. (1989) [47]	Case report	Moderately low
Kunz, U.(1988) [48]	Cohort	Moderately low
Kwak, Y. (2013) [49]	Cohort	Moderately low
LaCour, F. (1978) [50]	Case report	Moderately low
Lee, J. (2023) [51]	Cohort	Moderately low
Lee, Y. (2016) [52]	Case report	Moderately low
Li, B. (2021) [53]	Case report	Moderately low
Liu, B. (2019) [54]	Case report	Moderately low
Liu, Z. (2014) [55]	Case report	Moderately low
Lodin, J. (2019) [56]	Case report	Moderately low
Lohani, S.(2013) [57]	Case report	Moderately low
Marnat, G. (2023) [58]	Case report	Moderately low
Mayordomo, J. (2009) [59]	Case report	Moderately low
Mayr, U. (1982) [60]	Case report	Moderately low
McNeil, S. (1987) [61]	Case report	Moderately low
Meshkini, A. (2014) [62]	Cohort	Moderately low
Molloy, C. (1991) [63]	Case report	Moderately low
Mori, K. (2002) [64]	Case report	Moderately low
Morbée, L.(2015) [65]	Case report	Moderately low
Nadi, M. (2017) [66]	Case report	Moderately low
Naqvi, I. (2016) [67]	Case report	Moderately low
Nguyen, B. (2023) [68]	Case report	Moderately low
Sener, R.N. (1997) [69]	Case report	Moderately low
Ochi, M. (1996) [70]	Case report	Moderately low
Offiah, C. (2006) [71]	Case report	Moderately low
Oka, Y. (1994) [72]	Case report	Moderately low
Page, A. (1988) [73]	Case report	Moderately low
Patel, R. (2013) [74]	Case report	Moderately low
Poirriere, A. (2004) [75]	Case report	Moderately low
Prabhu, V. (2002) [76]	Case report	Moderately low
Puente, M.A. (2022) [77]	Cohort	Moderately low
Rajeev, N. (2024) [78]	Case report	Moderately low
Rashid, S. (2016) [79]	Case report	Moderately low
Rogers, M. (1990) [80]	Case report	Moderately low
Rogers, A. (2016) [81]	Cohort	Moderately low
Sayer, F. (2022) [82]	Case report	Moderately low
Shrestha, R. (2014) [83]	Case report	Moderately low
Slaviero, F. (2008) [84]	Case report	Moderately low
Sonnet, M. (2014) [85]	Case report	Moderately low
Choi, S.J. (2017) [86]	Case report	Moderately low
Szczygielski, J.(2021) [87]	Case report	Moderately low
Takayasu, T. (2012) [88]	Case report	Moderately low
Tinois, J. (2020) [89]	Case report	Moderately low
Tsitsopoulos, P. (2008) [90]	Case report	Moderately low
Tsuzuki, N. (2003) [91]	Case report	Moderately low
Van Der Meché, F. (1983) [92]	Case report	Moderately low
Varma, T. (1981) [93]	Case report	Moderately low
Wester K. (2008) [94]	Cohort	Moderately low
Wright D. (2015) [95]	Case report	Moderately low
Wu, X. (2018) [96]	Cohort	Moderately low
Xu, J. (2023) [97]	Cohort	Minimally low
Yaldiz, C. (2016) [98]	Case report	Moderately low
Yilmaz C. (2007) [99]	Case report	Moderately low
Yüksel M. (2016) [100]	Case report	Moderately low
Yokoyama, K. (1989) [101]	Case report	Moderately low
Zeng, T. (2011) [102]	Case report	Moderately low
Zhang, H. (2007) [103]	Case report	High
Zhao, H. (2023) [104]	Cohort	Moderately low

**Table 2:** Summary of Arachnoid Cysts Data.

Subarachnoid Cyst	Bleeding	Not Bleeding
**Number of participants**	331	1030
**Gender-Males**	79.15% (262)	76.12% (785)
**Age-mean**	8.73 years	6.98 years
**Diameter ^1^**	5–7 cm: 94.55% >7 cm: 0.9% <5 cm: 4.55%	3–4.5 cm: 98.9% >4.5 cm: 1.1%
**Type of bleeding**		Data not applicable
○ Subdural hematoma	○ 54.4% (180)	
○ Intracystic bleeding	○ 16.6% (55)	
○ Both	○ 29% (96)	
**History**		
○ Trauma	○ 36.25% (120)	○ 7.57% (78)
○ Sports	○ 10.6% (35)	○ 19.03% (196)
**Image**		
○ CT	○ 72.5% (240)	○ 44.37% (689)
○ MRI	○ 48.9% (162)	○ 37.96% (479)
**Treatment**		Data not available
○ Surgery	○ 84.59% (280)	
○ Conservative	○ 10.88% (36)	
**Side**		
○ Left	○ 55.59% (184)	○ 39.22%
○ Right	○ 28.70% (95)	○ 20%
○ Middle	○ 1.20% (4)	○ 2.04%
**Location**		
○ Middle cranial fossa	○ 73.12% (242)	○ 67.57% (696)
❖ Temporal	❖ 29.31% (97)	❖ 51.26% (528)
○ Posterior fossa	○ 1.20% (4)	○ 0.49% (5)
○ Sylvian fissure	○ 4.83% (16)	○ 1.07% (11)
○ Other	○ 20.85% (69)	○ 30.87% (318)

^1^ Diameter was not reported in all articles.

## Data Availability

All data available in the manuscript.

## Supplementary Materials

The following supporting information can be downloaded at https://www.mdpi.com/article/10.3390/jcm14196866/s1, PDF with search equation for each academic database.

## Abbreviations

The following abbreviations are used in this manuscript:

AC(s)	Arachnoid Cyst(s)
BVS	Biblioteca Virtual en Salud (Virtual Health Library)
CI	Confidence Interval
CSF	Cerebrospinal Fluid
CT	Computed Tomography
MRI	Magnetic Resonance Imaging
NHLBI	National Heart, Lung, and Blood Institute
OR	Odds Ratio
PRISMA	Preferred Reporting Items for Systematic Reviews and Meta-Analyses
PROSPERO	International Prospective Register of Systematic Reviews
