# Supplementary material for: Pediatric Arachnoid Cysts: A Comprehensive Systematic Review of Clinical Features and Therapeutic Approaches
Source: J Clin Med. 2025 Sep 28;14(19):6866. doi: 10.3390/jcm14196866 (PMC12525512; doi:10.3390/jcm14196866)
Supplement: Supplementary file 1 [file jcm-14-06866-s001.zip › jcm-3797389-supplementary.pdf]

Supplementary Material: Pediatric Arachnoid Cysts: A Comprehensive Systematic Review of Clinical Features and Therapeutic Approaches

1. Search Strategy

PubMed:
((Arachnoid Cysts) OR (Arachnoid Cyst) OR ("Arachnoid Cysts"[Mesh]) OR (Arachnoid Diverticula) OR (Arachnoid Diverticulas) OR (Leptomeningeal Cyst) OR (Leptomeningeal Cyst)) AND (("Hemorrhage"[Mesh]) OR (Hemorrhage) OR (Hemorrhages) OR (Haemorrhage) OR (Bleed) OR (Bleeding) OR (Bleeds))AND ((Pediatric) OR (Paediatric) OR (Pediatrics) OR ("Pediatrics"[Mesh]) OR (Child) OR (Children) OR (Childs) OR (Adolescent) OR (Adolescents))

Scopus:
TITLE-ABS-KEY (((Arachnoid Cysts) OR (ArachnoidCyst) OR (Arachnoid Diverticula) OR (ArachnoidDiverticulas) OR (Leptomeningeal Cyst) OR (Leptomeningeal Cyst)) AND ((Hemorrhage) OR (Hemorrhages) OR (Haemorrhage) OR (Bleed) OR (Bleeding) OR (Bleeds)) AND ((Pediatric) OR (Paediatric) OR (Pediatrics) OR (Child) OR (Children) OR (Childs) OR (Adolescent) OR (Adolescents)))

Web of Science:
((Arachnoid Cysts) OR (Arachnoid Cyst) OR (ArachnoidDiverticula) OR (Arachnoid Diverticulas) OR (Leptomeningeal Cyst) OR (Leptomeningeal Cyst)) AND ((Hemorrhage) OR (Hemorrhages) OR (Haemorrhage) OR (Bleed) OR (Bleeding) OR (Bleeds)) AND ((Pediatric) OR (Paediatric) OR (Pediatrics) OR (Child) OR (Children) OR (Childs) OR (Adolescent) OR (Adolescents))

BVS:
((Arachnoid Cysts) OR (Quistes Aracnoideos) OR (Arachnoid Cyst) OR (Quiste Aracnoideo) OR (Arachnoid Diverticula) OR (Diverticulos Aracnoideos)OR (Arachnoid Diverticulas) OR (Leptomeningeal Cyst)OR (Quiste Leptomeningeo) OR (Quistes Leptomeningeos) OR (Leptomeningeal Cyst)) AND ((Hemorrhage) OR (Hemorragia) OR (Hemorrhages) OR (Hemorragias) OR (Haemorrhage) OR (Bleed) OR (Sangrado) OR (Bleeding) OR (Bleeds) OR (Sangrados)) AND ((Pediatric) OR (Pediatrico) OR (Paediatric) OR (Pediatrics) OR (Pediatricos) OR (Pediatria) OR (Child) OR (Niño) OR (Children) OR (Niños) OR (Childs) OR (Adolescent) OR (Adolescente)OR (Adolescents) OR (Adolescentes))
